# Supplementary material for: Are carbon emissions trading and green financial instruments synergistic? -Comprehensive quantitative research based on content analysis
Source: PLoS One. 2024 Mar 7;19(3):e0298601. doi: 10.1371/journal.pone.0298601 (PMC10919601; doi:10.1371/journal.pone.0298601)
Supplement: S1 Data — (ZIP) [file pone.0298601.s001.zip › Data/地级市Z_F工作报告.zip!/╒π╜¡/╜≡╗¬╩╨/└┤╫╘╣1⁄2╓┌║┼í╛┬φ┐╦╩2╛▌═°í┐_2017.docx]

各位代表：

现在，我代表金华市人民政府向大会作政府工作报告，请予审议，并请市政协委员和其他列席人员提出意见。

一、本届政府及2016年工作回顾

　　市六届人大一次会议以来，在中共金华市委的坚强领导下，本届政府和全市人民一道，围绕“百姓富裕、浙中崛起”目标，按照“走在前列、共建金华”要求，认真落实市委各项决策部署和市六届人大各项决议，在赶超发展中砥砺前行。六年来，28项主要经济指标中，有24项全省占比提高；2016年全市实现生产总值3635亿元，年均增长8.9%；一般公共预算收入338亿元，年均增长12.7%；城乡常住居民人均可支配收入分别达到46554元和21896元，年均增长10.2%、11.3%。

（一）改革开放创新重大举措有效落地。市县两级“四张清单一张网” 基本建成，权力清单事项平均削减36.8%，取消行政事业性收费97项，率先探索“互联网+政务服务”建设，“网上政府”建设三年行动计划全面实施。供给侧结构性改革初见成效，40多项国家和省部级改革试点扎实推进。义乌国际贸易综合改革试点取得重大突破，国家现代服务业综合试点绩效居全国前列，全国低丘缓坡综合开发利用试点进展顺利，浦江协调推进“四个全面”战略布局试点成效明显。商事制度改革加快推进，新增市场主体数量居全省前列。认真落实国地税征管体制改革及“营改增”试点，“互联网+财税”做法在全省推广。科技创新力度加大，研发经费支出占生产总值比重超过2%。义乌开发区升格为国家级开发区。规划建设22个特色小镇，其中省级11个。累计入选和引进“****”专家129人、评选“双龙计划”人才项目154个。获评中国“一带一路”最具活力城市，义甬舟开放大通道加快建设，“义新欧”班列开通线路8条、运送量突破1万标箱，义乌国际邮件互换局业务量居全国第四，金义综合保税区通过国家验收，全球跨境电商大会永久落户，陆港、口岸加快建设，海外名校学子走进金华古村落活动产生良好影响。外贸出口额年均增长25.2%，总量跃居全省第三。对口帮扶工作扎实有效。

（二）经济转型升级取得新进展。三次产业结构实现“二三一”向“三二一”转变，三产增加值占生产总值比重达52.3%，规上工业总产值突破5000亿元，工业增加值突破1000亿元，农业全省排名实现晋位。先进装备制造业加快发展，新能源汽车产业规模居全省第三，新引进华科汽车、华灿光电等总投资超15亿元的浙（婺）商回归重大产业项目38个，新增上市公司10家、新三板挂牌企业46家，“金华制造上京东”企业6277家，“机器换人、空间换地”经验在全省推广。义乌进入生产总值千亿元俱乐部。永康规上工业总产值突破千亿元，成为全国首个县级质量强市示范城市。兰溪规上工业进入全省20强，华东铝业实施战略重组。浦江水晶等行业治转结合成效明显。武义获批国家级出口质量安全示范区。东阳建筑业发展稳居全国县市榜首。网络零售额居全省第二，7个县市全部入围中国电子商务百强县，青岩刘等一批中国淘宝村蓬勃发展。旅游收入居全省第四，横店影视文化产业实验区成为国家级文化和科技融合示范基地，影视城年游客接待量居全国景区前三。金融存贷款余额位居全省第四。现代农业加快发展，连续获评全省粮食生产先进市，建成省级粮食生产功能区81万亩、省级现代农业综合园区11个、设施农业21万亩，培育规范性农民专业合作社812家、示范性家庭农场251家。金华国家农业科技园区获批。兰溪、浦江入选全国休闲农业和乡村旅游示范县。

（三）环境综合治理走在前列。全国水环境综合整治、违法建设治理、农村生活垃圾分类及资源化利用等现场会在我市召开，成功创建国家园林城市、国家森林城市、国家节水型城市，各县市创建为国家卫生城市、省级森林城市，磐安、义乌成为国家生态县市。全省“五水共治”第一枪在浦江打响，河长制、“治水十法”在全国推广，农村面源污染治理成效明显，沙金兰等水源涵养生态功能区建设深入推进，兰江等全流域防洪能力进一步提升，兑现了“一年灭黑臭、两年提水质、三年可游泳”承诺，地表水Ⅲ类以上水质断面占比达到97.6%，连续三年荣获大禹鼎。实施气尘合治三年行动，2016年金华市区PM2.5平均浓度比2013年下降34.3%，空气质量优良率提高到80.6%，改善幅度居全省前列。全面推进“三改一拆”“整治环境、靓化金华”等行动，婺城二七棚户区改造成全省典型，金东横塘沿城中村改造做法获百姓点赞，东阳红椿巷、兰溪排岭、浦江金狮湖、义乌仓后等区块大拆大整取得突破。深化美丽乡村建设，加强历史文化村落保护利用，农村生活垃圾“两次四分法”在全国推广，垃圾分类处理和污水治理村庄覆盖率分别达98.1%和95%。浦江成为全省美丽乡村示范县，浦阳江国家湿地公园获批。

（四）都市区共建加快推进。编制完成都市区规划纲要和综合交通规划、轨道交通线网规划、金义都市新区规划、基础设施空间一体化规划等8个专项规划，村庄规划覆盖率达95%。实施都市区建设三年行动，基本形成“一小时通勤圈”，城市化率达到65.7%，被列为长三角26个中心城市、沪杭金发展带重要节点城市。金华跨入高铁时代，杭长客专、金温铁路新线开通，金义东市域轨道交通、金甬铁路开工建设，金华铁路南站和新货场投入运营，铁路客站交通枢纽区改造加快推进。东永高速、杭金衢高速改扩建一期和一批城际快速路建成通车，义乌疏港高速、杭绍金台高速、临金高速加快建设。布局推动金义都市新区、金华山旅游经济区、多湖中央商务区开发建设，湖海塘等重点区块建设步伐加快。开通BRT 线路6条，金华市区公交分担率提高到20.2%。衢江金华段航运开发和义乌机场改扩建、横店通用机场建设加快推进。治堵工作考核三年全省第一。九峰水库验收运行，第二水厂建成使用。我市成为特高压交流直流混联城市，全社会用电量突破300亿千瓦时。

（五）民生福祉持续改善。财政民生支出占比超过70%，公共服务均等化程度达90%以上。每年办好十方面实事。全面消除年人均收入4600元以下贫困现象，城乡居民收入差距缩小。武义、磐安实现“摘帽快跑、绿色发展”。新增城镇就业37万人，城镇登记失业率控制在2.9%以内。最低工资标准提高69%，企业退休人员基本养老金标准提高57%，城乡居民基础养老金最低标准提高100%，农村最低生活保障标准提高18.5%。建成城乡居家养老服务照料中心3020家，惠及20余万老年人。金东居家养老模式获评全国民生示范工程。城乡居民医疗保险实现并轨。基本公共文化服务体系不断健全，建成农村文化礼堂784个、居全省第四，市体育中心、中国婺剧院、东阳木雕博物馆等投入使用。开展最美系列创建评选，一城五人“感动中国”。教育改革发展步伐加快，义务教育入学率和巩固率达100%，实现全国义务教育基本均衡县和省体育强县全覆盖。“双下沉、两提升” 扎实推进，9家县级医院与上级医院开展紧密型合作办医，基本形成城乡20分钟医疗服务圈。依法稳妥实施全面两孩政策，出生人口性别比实现六连降。社会治安、安全生产、食品药品安全、信访、调解、社区矫正、应急管理等工作扎实有效，实现平安银鼎全覆盖，婺城、武义、磐安获平安金鼎。创建8890便民服务平台，为群众解决各类诉求162万件，办结率和回访满意率99%以上。

　（六）政府自身建设不断加强。从严落实中央八项规定精神，扎实开展党的群众路线教育实践活动、“三严三实”专题教育、“两学一做”学习教育，认真抓好中央和省委巡视组反馈意见的整改落实。建立健全政府常务会议学法、领导干部夜读学法、政府法律顾问等制度，行政诉讼案件败诉率明显低于全省平均水平。坚决贯彻市委决策部署，主动接受人大及其常委会依法监督、政协民主监督和社会监督、舆论监督，邀请人大代表、政协委员列席政府常务会议。改进办理方式，认真做好人大代表、政协委员建议提案办理工作。加强廉政风险点防控，审计监督实现全覆盖，政务公开力度进一步加大。建立政府投资项目超概算责任追究办法、财政性资金竞争性存放管理制度，强化公共资源交易领域信用建设，完成党政机关公车改革，“三公”经费支出持续下降。深化行政执法体制改革，综合行政执法全面推进。组建市场监管局、卫生计生委。

全面发展老龄、残疾人和关心下一代等事业，广泛开展慈善救助活动。支持工会、共青团、妇联等人民团体开展工作。加强民族、宗教、外侨、对台、统计、气象、民防、档案、地方志、仲裁等工作。完成“六五”普法、第三次经济普查和第六次人口普查，启动“七五”普法。支持国防建设和军队改革，实现全国双拥模范城“三连冠”。金华在全省首批获得地方立法权。

2016年是本届政府的收官之年。我们积极应对经济下行压力，扎实推进供给侧结构性改革，坚定不移打好转型升级系列组合拳，全面完成年初确定的工作目标，实现了“十三五”良好开局。

这一年，我们全力服务保障G20杭州峰会。举全市之力实施“四大计划、十大行动”，兑现了金华的庄严承诺，圆满完成了省委、省政府交给我市的工作任务。

这一年，我们大力发展实体经济。全面落实市委关于补短板的决策部署，开展专题调研，出台“1+8+X”政策体系，推进“五个一百”行动，加快发展五大千亿产业，加大企业“两链”风险化解和帮扶工作力度，开展“三百竞赛”活动，重大产业项目投资增长29%、高新技术产业投资增长18.2%，新引进吉利英伦、零跑科技等总投资超15亿元重大产业项目12个，浙（婺）商回归资金332亿元，列入国家首批消费品工业“三品”战略示范试点，大企业大集团产值（营收）增速连续11个月保持10%以上。金华科技城入驻企业62家、引进科研院所2家。菜鸟电商园建成运营，腾讯众创空间落户。美保龙、九峰牧场等成为全省畜牧业转型升级典型。

这一年，我们合力加快都市区建设。着力规划共绘，加快推进“多规合一”，启动编制“三条廊道”专项规划和都市区建设第二个三年行动计划。着力设施共建，固定资产投资增长13.5%，重大基础设施投资增长37%，完成交通基础设施投资220亿元，金义东市域轨道交通项目试验段开工建设。着力生态共保，“五水共治”“三改一拆”工作考核居全省前列，河塘清淤量全省第一，东阳“环境革命”、磐安“新农村乡风革命”等掀起城乡环境整治新高潮，横店成为小城镇风貌提升全省样板。

这一年，我们强力推进供给侧结构性改革。开展金华开发区、永康供给侧结构性改革省级试点，扎实推进 “三去一降一补”，处置“僵尸企业”102家，淘汰落后产能企业103家，整治关停“低小散”企业3206家，商品住宅去化周期降至10.7个月，为企业减负84亿元，处置不良贷款力度全省最大。

这一年，我们加力对接国家“一带一路”倡议。制定实施三年行动计划，“义新欧”班列新增6条线路，金义综合保税区签约入驻企业40家。“引进来”和“走出去”并举，实际利用外资3.5亿美元，对外直接投资8.3亿美元、承包工程营业额4亿美元。11家跨境电商园区、14家公共海外仓列入省级试点，居全省第一。

这一年，我们着力提高群众获得感。财政民生支出增长13.2%，占全部支出的73.6%，十方面民生实事全面完成。全体居民可支配收入增长8.1%，低收入群众收入增长18.9%。新增城镇就业7.8万人，城乡居民养老保险和医疗保险参保率分别为95%、98%。义务教育标准化学校覆盖率达95%，金东、义乌通过省教育基本现代化县验收。实现优质医疗资源下沉乡镇全覆盖，分级诊疗和家庭责任医生签约服务加快推进，浙医四院建成运营，市妇幼保健院、中心血站迁建项目开工建设。郑义门、延福寺等一批文保单位得到修缮，浦江上山文化遗址公园落成，市科技文化广场加快建设。完成文化惠民“五进五送”4.5万场。群众性体育活动广泛开展，重点公共体育场所向社会开放。

各位代表，发展成绩来之不易。这是省委、省政府和市委坚强领导的结果，是市人大、市政协和社会各界监督支持的结果，是全市人民创业创新、奋力拼搏的结果。在此，我代表市人民政府，向全市人民和外来投资者、建设者，表示衷心的感谢！向人大代表、政协委员，向各民主党派、工商联、人民团体、无党派人士，向驻金部队、武警官兵、部省属驻金单位，表示衷心的感谢！向所有关心、支持我市建设和发展的港澳台同胞、海外侨胞以及社会各界朋友，表示衷心的感谢！

我们也清醒地看到，我市经济社会发展和政府工作还存在一些不足和问题：县域经济向城市经济、都市区经济转型步伐不快，破解“散”这个战略性短板任重道远；科技创新能力和高新技术产业发展水平不高，传统产业改造提升亟待突破；一些企业转型升级面临困难较大，防范化解企业“两链”风险任务艰巨；重点领域和关键环节改革系统集成不够，“放管服”改革还需深化；基本公共服务供给仍然不足，安全生产、食品安全、网络安全、社会治安还存在一些薄弱环节。同时，一些政府工作人员不担当、不作为问题没有根本改变，“四风”问题面临反弹压力，消极腐败现象时有发生，廉政建设任务依然艰巨。对此，我们一定采取更加有力的措施加以解决。

二、今后五年的目标任务和工作重点

　　市第七次党代会描绘了今后五年发展的宏伟蓝图，我们要按照市委的总体部署，干字当头、狠抓落实，扛起改革发展大旗，加快推进浙中崛起，充分发挥金华在浙江中西部崛起中的龙头、带动、示范作用。

今后五年工作指导思想是：高举中国特色社会主义伟大旗帜，以马克思列宁主义、毛泽东思想、邓小平理论、“三个代表”重要思想、科学发展观为指导，全面贯彻党的十八大和十八届三中、四中、五中、六中全会精神，深入学习习近平总书记系列重要讲话精神，统筹推进“五位一体”总体布局和协调推进“四个全面”战略布局，以“八八战略”为总纲，全面贯彻落实市第七次党代会精神，围绕“全面小康、浙中崛起”总目标，按照 “走在前列、共建金华”要求，大力实施群城聚市、实业兴市、创新强市、开放活市、环境立市五大战略，着力打造全省高质量发展的重要增长极，确保与全省同步高水平全面建成小康社会，加快建设圈带聚合、协同共兴、富有活力、和谐宜居的现代化都市区。

建议今后五年的发展目标是：主要经济指标增速高于全省平均水平，到2021年生产总值超过5000亿元，一般公共预算收入超过500亿元，城乡常住居民人均可支配收入分别达到6.5万元和3.2万元。

（一）聚焦群城聚市，加快共建都市区

　　完善都市区“同心圆”规划。健全规划体系，推进“多规合一”，编制实施都市区规划，完成新一轮城市总体规划修编，统筹做好“三条廊道”等重点专项规划，编制城镇控制性详细规划，实现重点区域城市设计全覆盖，加强城市天际线、主色调、疏密度、景观带和显山露水视线通廊的建设引导。

建设网络状城市群、组团式都市区。建立健全都市区共建共融共享共赢体制机制，加大对都市区重大区块、重大平台和重大项目的统筹协调力度，实施都市区建设第二个三年行动计划，提升都市核心区首位度，促进都市区融合发展。婺城、金东作为中心城市的核心区，要高起点规划、高标准建设、高水平管理，进一步做优老城区、做强新城区、做美大郊区。金华开发区要加快打造工业发展的主引擎、城市拓展的核心区、产业转型的新高地。金义都市新区要加快打造高端产业集聚、科技创新驱动、对外开放引领的都市区聚合发展战略支点。金华山旅游经济区要打响“三教名山”“双龙胜景”“休闲养生”品牌，加快建设现代旅游休闲区。多湖中央商务区要加快建设“五大中心”，打造浙中城市建设标杆区。湖海塘、尖峰山、铁路客站、二七区块、婺州古城、石门农场、三江六岸、高铁南站新组团等重点功能区要加快建设步伐，打造城市发展特色亮点。义乌要勇立潮头、勇扛旗帜、勇当标兵，建设世界小商品之都。兰溪建设产业振兴示范区，东阳建设影视旅游名城，永康建设现代化国际五金名城，浦江建设世界水晶产销中心，武义建设温泉名城养生胜地，磐安建设江南生态养生源。

合力构筑“三条廊道”。围绕建设全国性综合交通枢纽目标，共建综合交通廊道，完成投资1500亿元以上，建成5条高标准铁路、5条高速公路、5条城际快速路，形成轨道交通、高速公路、快速干线复合闭合圈，实现县县通高铁。依托金义主轴，共建金义科创廊道，重点建设“一城一区”高能级科创平台，布局科技园区、高教新区、特色小镇等科创载体，集聚一批大院大所和高精尖人才，打造引领都市区发展的科技创新带、高新产业带、品质生活带和科技人才高地。按照“一年打基础、三年见成效、五年成廊道”要求，共建浙中生态廊道，推进全流域综合治理，加快生态河流、美丽城防、绿道联网、彩色林带、休闲公园等建设，实施连片造田造地7万亩，打造山水林田湖生命共同体，完成投资2600亿元以上。

统筹城乡协调发展。加强城市规划、建设和管理工作，加快智慧城市、海绵城市、精品城市、公交都市建设步伐。抓住“三改一拆”这一“牛鼻子”，坚持以改造带项目，能改则改、能快则快，加大城中村连片改造力度。加快城市有机更新，加大背街小巷、老旧小区改善力度，加强历史文化街区保护。加强地下综合管廊建设，提升城市基础设施和配套设施水平。积极实施“小县大城”建设，培育发展小城市、中心镇和特色小镇，保护传统村落，推进产业非农化、农民市民化、城乡一体化，城市化率力争超过全省平均水平。加快打造既有现代文明、又有田园风光的美丽乡村升级版，全域推进农村人居环境整治和基本公共设施建设，美丽乡村“三个标配”达到98%以上，争创省级美丽乡村示范县4个、示范乡镇45个、示范精品村150个。支持磐安国家级重点生态功能区建设，提升发展金磐扶贫开发区。进一步改善武义柳城、兰溪水亭等民族乡镇生产生活条件。

（二）聚焦实业兴市，全力振兴实体经济

　　打造全省先进制造业基地。大力实施“十百千万”工程，积极创建中国制造2025试点城市、全国质量强市示范城市和国家消费品工业“三品”战略示范试点城市，加大“四换三名” “互联网+”“机器人+”“标准化+”工作力度，先进装备制造业规上总产值突破4000亿元，新能源汽车产业形成150万台整车生产能力和超千亿元产业规模，新增产值超百亿元大企业10家、超十亿元企业100家、中国驰名商标10个、省著名商标200个、省名牌产品50个，战略性新兴产业增加值占规上工业增加值比重达28%，高新技术产业占比达38%。依托“三条廊道”做大平台，形成重大项目生成机制，全力招大引强、招才引智、招院引所，新引进投资50亿元重大项目50个、企业总部50个、高校2所、科研院所30家。同时，加大“个转企、小升规”工作力度，新培育市场主体30万家以上。

加快发展现代服务业。深化国家现代服务业综合试点，加快11个省级服务业集聚示范区建设。以电子商务、互联网泛娱乐和大数据产业为重点，大力发展信息经济，力争五年达到万亿级规模。发展壮大文化影视时尚产业，五年产值（收入）超过5300亿元。加快推进全域旅游，培育“千万十亿”大景区，新增国家5A级景区2家以上、4A级景区20家、3A级景区40家，旅游总收入实现五年翻番。加快构建万亿级市场体系，鼓励龙头市场加快营销网络布局，大力支持菜鸟、京东等电商平台建设，发展壮大“义乌购”“尚五金”“东作云”等线上平台，重视办好品牌展会，巩固提升浙中商贸中心地位。积极引导企业对接多层次资本市场，新增股份制企业500家、上市挂牌企业300家，力争直接融资比例达到10%，金融存贷款余额双双突破万亿元。同时，大力发展总部经济、楼宇经济、廊道经济和都市经济。

深入推进农业供给侧结构性改革。全面落实粮食安全责任制，推进“互联网+农业”，加强农产品品牌建设，建设绿色高效生态农业强市。到2021年，建成省级农业产业集聚区3个、特色农业强镇10个，种子种苗生产基地1万亩、设施农业25万亩，市级以上农业龙头企业达300家、示范性家庭农场400家、上市企业10家。大力推进畜牧业转型升级，建成省级美丽牧场100家、现代生态循环农业示范区10个、示范主体100个以上。积极发展创意农业、休闲农业、景观农业、养生农业，创建市级农旅融合示范点100个、国家级休闲农业与乡村旅游示范点5个。

　（三）聚焦创新强市，再造体制机制新优势

　　增强科技推动力。完善区域创新体系，深入实施“****” “双龙计划”“八婺英才计划”“青年创业谷计划”，推进大众创业、万众创新，跻身国家创新型试点城市行列，争创国家自主创新示范区。到2021年，实施自主创新重大专项100个、创建高新技术产业园区3个，国家高新技术企业达900家、省级科技型中小微企业3000家、市级以上研发机构1300家，专业技术人才超过60万人，研发经费支出占生产总值比重达2.6%。

提升平台承载力。加快打造“一城一区”科创大平台，整合提升“多园多镇”，争创国家级高新技术产业开发区。做优做强金华、义乌国家级开发区，支持永康、东阳、兰溪开发区创建国家级开发区，做新做美省级产业集聚区、开发区、工业园区，做专做精特色小镇、工业强镇、电商新镇，做活做特科技孵化器、众创空间。到2021年，特色小镇累计投资超1000亿元，培育省十强工业强镇2个、电商新镇50个。

激发改革新活力。以共建“三条廊道”为契机，积极探索建设运营管理的投入分担机制和收益分成机制。以供给侧结构性改革为主线，全面推进“三去一降一补”。充分发挥国家和省部级改革试点示范引领作用，积极争取更多改革试点。支持义乌建设国际贸易改革试验区、浦江打造“四个全面”战略布局标杆县，统筹推进各县（市、区）重点改革。支持国防建设和军队改革，积极推进军民融合深度发展，创建省级军民融合创新示范区。联动推进土地、户籍、产权、社保、撤村建居等城乡综合配套改革。

　（四）聚焦开放活市，勇当“一带一路”排头兵

　　建设国际商贸高地。深化义乌国际贸易综合改革试点，建立健全与小商品进出口相适应的交易平台、贸易通道和监管方式，提升贸易便利化水平。充分发挥金义综合保税区、义乌B型保税物流中心功能作用，做强金义跨境通、义乌国际邮件互换局等开放平台，争取设立金义小商品自由贸易港区。推进义乌国际陆港与宁波舟山港一体化运行，加快推进浙中公铁水联运港、兰溪嘉宝物流港、东阳国际物流港、永康无水港等陆港建设，打造国际货物集散地。

拓展开放通道。加快义甬舟开放大通道建设，实施金甬铁路等100个重大项目，完成投资3500亿元，全面融入义甬舟交通带、物流带、产业带、科创带建设，加快构建“海上丝绸之路”。完善“义新欧”班列市场化常态运行机制，建设经贸合作园、物流分拨中心，加快拓展“陆上丝绸之路”。加快跨境电商海外仓布局建设，争取设立国家跨境电商综合试验区，积极开拓“网上丝绸之路”，跨境电商进出口年均增长30%以上。

构筑开放新格局。厚植小商品出口优势，扩大高新技术产品、名优特产品出口，补齐进口、加工、转口贸易短板，大力发展跨境电商、市场采购等新型贸易，对“一带一路”沿线国家出口年均增长10%，到2021年占全市出口半壁江山。优化外资营商环境、产业结构、区域布局和招引方式，提高利用外资规模质量，实现五年翻番、累计达到27亿美元。加快推动外经发展，支持企业集团式、产业链式“走出去”投资办厂、海外营销和开展跨国并购。深化对外合作交流，充分利用侨务、友城、海外商会等资源，加强经贸、科技、环保、文化等领域合作，建好中欧生态工业园、中欧智造园、中捷水晶产业合作园等。

（五）聚焦环境立市，持续增进民生福祉

　　优化政务服务。加快推进“最多跑一次”改革，从与企业和人民群众生产生活关系最紧密的领域和事项改起，全面梳理政务服务事项，实现受理一窗化、服务集成化、全程电子化，让数据多跑腿，群众少跑腿、争取不跑腿，提高“放管服”改革实效，全力打造最优政务服务环境，建设“互联网+政务服务”先行区，让百姓办事更便利、让企业办事更省心。

优化自然生态。严格执行《金华市水环境保护条例》，深入实施美丽金华建设行动，决不把违法建筑、污泥浊水、脏乱差带入全面小康。坚决打好治水治气、治危拆违、治城治乡、治土治山系列攻坚战，全面推行污水处理“金华标准”，深入实施城乡生活垃圾分类处理和废弃物处置，全面开展土壤污染防治行动，创建森林城市群和省级生态文明示范区、美丽浙江先行区。两年基本完成城乡危房治理，三年完成小城镇环境综合整治，四年基本消灭城中村，五年全流域水质达到或优于Ⅲ类标准、城市空气质量优良率达85%、PM2.5平均浓度比2013年下降40%、森林覆盖率稳定在61%以上、污染耕地和污染地块安全利用率达91％。

优化公共服务。加大民生投入，基本公共服务均等化程度达到95%以上。精准扶贫帮困，大力推进旅游、电商、光伏扶贫，加大异地搬迁、结对帮扶力度，确保全面小康一个不少、一户不落。织密扎牢就业社保安全网，城镇登记失业率控制在3.5%以内，积极推进社保扩面工作，实现居家养老服务全域化，提高住房保障水平。推进教育现代化，高标准普及15年教育，重视发展职业教育、高等教育和特殊教育、老年教育、家庭教育，筹建高水平应用型高校。深化医药卫生体制综合改革，提高“双下沉、两提升”实效，完善分级诊疗机制，建设“健康金华”。加强“信义金华”建设，大力发展文化体育事业，开展最美系列创建评选，争创全国文明城市，建好管好用好农村文化礼堂。鼓励社会资本投资文化、教育、医疗、养老等事业。

优化社会治理。加强平安金华、法治金华建设。打造社会治安防控升级版，依法防范和惩治各类违法犯罪。牢牢守住安全生产底线。积极创建国家食品安全城市、全国网络监管服务示范城市。实施重要地质灾害隐患点三年清零行动。提高防灾减灾、应急管理能力，做好重大事项社会稳定风险评估工作。积极开展双拥共建，深化8890便民服务、12345政务服务，建好乡镇（街道）“四个平台”，加强和创新信访、调解、社区矫正、“七五”普法等工作，确保人民安居乐业。

三、2017年主要工作

　　建议今年经济发展主要预期目标为：生产总值增长7.5%左右，城乡居民收入、财政收入增长与经济增长同步，供给侧结构性改革取得新进展，节能减排降碳指标完成省定目标任务。

　（一）以“最多跑一次”改革撬动各项改革落地生根

　　全力推进“最多跑一次”改革。分批公布“最多跑一次”目录，确保年内覆盖80%以上的政务服务事项。推进简政放权和一窗化受理，建立事项受理容缺机制，形成覆盖行政许可、行政征收、行政裁决、行政服务等领域的一次办结机制。推行“双随机、一公开”，形成事中事后监管一次到位机制。推行淘宝式全流程网上办理模式、移动端APP应用，尽快联通各部门信息数据，形成 “互联网+政务服务”零上门机制。

整体推进行政审批制度改革、商事制度改革、政事分开改革和事业单位改革，实现投资项目网上审批、核准、备案，规范和培育中介服务市场，推进 “最多跑一次”改革向中介机构延伸。

扎实推进重点领域和关键环节改革。支持义乌改革发展和浦江“四个全面”战略布局标杆县建设，加快推进东阳国家级文化和科技融合示范基地建设，深化金华开发区和永康供给侧结构性改革试点，扎实开展兰溪海绵城市、武义低碳城市、磐安林业股份制改革等省级试点。

　（二）以新旧动能接续转换加力发展实体经济

　　实施五大千亿产业三年行动。加快构建5+X现代产业体系，确保网络零售额、跨境电商零售出口额、软件与信息服务业营业收入均增长20%，先进装备制造业产值增长8.5 %，健康生物医药产业增长10%，旅游业收入增长18%，文化影视时尚产业增长10%，争取设立金华国家数字内容产业基地和浙江（金华）网络文化产业试验区。

实施传统产业“老树发新芽”行动。制定实施水晶、五金、建材、化工、小商品、纺织服装、红木家具、建筑装配、批发零售等传统产业改造提升行动计划，推进火腿、婺州窑、木雕、茶叶、酥饼、黄酒、中药材等历史经典产业提升发展。落实大企业大集团、高成长企业培育和小微企业成长计划，新增产值超50亿元企业5家、股份制企业100家、上市挂牌企业60家，新增入驻京东商城制造企业2000家、工业机器人1000台，为企业减负100亿元，入选浙江制造精品和省级优秀新产品15个、省名牌产品10个、省著名商标40个。

实施招大引强扩投资行动。引进浙（婺）商回归资金350亿元、内资420亿元、实际利用外资4.3亿美元，引进投资超50亿元大项目10个以上，确保固定资产投资增长12%，工业投资增长7%，重大基础设施项目、重大产业项目、重点技改项目、生态环保投资均增长17%以上。

（三）以“三条廊道”建设推进都市区共建共融

　　编制都市区规划，推进新一轮城市总规修编，统筹做好“三条廊道”专项规划，强化规划引领和依法管控。

加快建设综合交通廊道。完成交通投资260亿元，金义东市域轨道交通项目全线开工，加快建设杭温、金台、金甬、金建铁路和临金、杭绍金台、义乌疏港等高速公路，开工建设义东永高速、杭金衢高速改扩建二期和金义中线、G351国道，加快建设金武、金兰、义永、义兰等城际快速路。衢江航运金华段建成通航。加快义乌机场改扩建步伐，建成横店通用机场。优先发展公共交通，新增停车位3000个。

加快建设金义科创廊道。推进科创平台建设，积极创建国家高新技术产业开发区，金华科技城引进大院大所1家、研发机构5家、产业化项目10个以上，支持建设菜鸟商学院、吉利衡远新能源研究院。完善科技政策，创新科技金融，制定实施科技创新龙头企业、高新技术企业、科技型中小微企业 “三倍增”计划，推进百强企业研发机构和规上企业创新活动“两个全覆盖”，做大做强创新主体。

加快建设浙中生态廊道。按照“一年打基础、基本形成廊道主体框架”的要求，开工建设653个重点项目，投资470亿元，加固病险水库19座，新开工建设河道堤防53公里，新增截污纳管400公里。启动实施市区三江六岸景区贯通和景观提升工程，还绿于民、还景于民，打造浙中生态廊道的示范样板。

　（四）以科技创新提升转型发展驱动力

　　建设科创平台和载体。加快建设金华科技城、义乌科创新区，支持北大信息科技园扩容，加快中科院金华科技园、浙中信息产业园、金东信息软件创业园、浙师大婺城科创园、永康五金生产力促进中心、武义科技城等科技园区建设。推进金华开发区新能源汽车、永康现代农业装备、兰溪光膜新材料、浦江水晶等特色产业园区建设。加快特色小镇建设，完成固定资产投资210亿元。鼓励利用闲置厂房建设科技孵化器、众创空间。

推进科技成果产业化。建立10亿元以上的科创投资基金，支持企业加大科技投入，加强研究开发，强化知识产权保护，与大院名校加强产学研合作，推进国家标准化综合改革试点，新增国家高新技术企业100家、省级科技型中小企业470家，实现万人发明专利拥有量7.5件、重大发明专利产业化项目100个、技术市场交易额15亿元以上，创建国家知识产权示范城市。

加快集聚创新人才。全面落实人才新政，重视企业家队伍建设，大力培育战略型、领军型企业家和新型职业经理人，加强高层次人才、高技能人才和工匠队伍建设，培养引进“****”10人、“双龙计划”人才项目30个以上。

（五）以大拆大整打赢环境综合治理攻坚战

　　坚持有违必拆，联动打好治危拆违、小城镇环境综合整治攻坚战，整治环境、靓化金华。完成全部D级危房和涉及公共安全的C级危房治理，实施城中村、景中村改造1000万平方米，旧厂区改造300万平方米，旧住宅区改造300万平方米以上，拆除违法建筑1000万平方米，小城镇环境综合整治完成40%以上，创建省“无违建县（市、区）”1个、“基本无违建县（市、区）”2个。推进城镇低效用地再开发7000亩、盘活存量用地8500亩。

坚持有污必治，横下一条心，坚决打赢剿灭劣Ⅴ类水攻坚战。按照“干流水质全达标，支流溪流灭Ⅴ类，沟渠河塘荡污浊”的年度目标，全面推进截污纳管、小微水体整治、入河排污口整治规范、工业污染整治提升、农村双治改造提升、农业面源污染治理提升、沟渠河塘清淤修复和智慧治水保障等“八大工程”。同时，打好蓝天保卫战，努力减少重污染天气，全面完成高污染燃料锅炉淘汰改造任务，全年PM2.5平均浓度比2013年下降35%、空气质量优良率达到81%以上。深入开展土壤污染防治行动，农业“两区”内土壤清洁率达90%以上，重点企业工业危险废物无害化处置率达到95%。

坚持有劣必除，重视防范化解企业“两链”风险，坚决淘汰落后产能。完成处置“僵尸企业”30家、淘汰落后产能企业80家、整治提升“低小散”企业1000家。提升金融服务实体经济能力，严厉打击逃废债行为，确保不发生系统性金融风险，新增融资规模700亿元以上。综合施策强化保障性住房体系建设，多措并举促进房地产市场持续健康发展。

（六）以义甬舟大通道建设提升对外开放水平

　　加快建设义甬舟开放大通道。大力实施80个重大支撑项目，完成投资400亿元。加强金义综合保税区封关运行和招商工作，积极推进二期项目建设。加快实现义乌国际陆港与宁波舟山港 “同港同关同检”，推进浙中公铁水联运港二期建设，提升兰溪嘉宝物流港、东阳国际物流港、永康无水港功能。加快建设中欧生态工业园、中欧智造园、中捷水晶产业合作园。

提高国际贸易便利化水平。深化义乌国际贸易综合改革试点，做优一般贸易，扩大服务贸易，发展加工贸易，探索建立外贸综合服务平台，推进“单一窗口”模式，加快建设省级跨境电商创新发展示范区。确保外贸出口增长6%，对“一带一路”沿线国家出口额超过1500亿元，进口增幅同比提高10个百分点，服务贸易进出口增长10%。

深化对外交流合作。深入实施对接“一带一路”倡议三年行动计划，加强经贸、文化、科技、教育等各领域交流合作。支持企业“走出去”开展跨国并购，建立产销网络，组织参加境内外展会100个以上。继续办好海外名校学子走进金华古村落等活动。积极参与沪杭金发展带建设，加强与海西经济区、四省九方经济协作区互动，做好东西部扶贫协作和对口支援帮扶工作。

（七）以“三转”促进“三农”工作

　　加快农业转型。全面落实粮食安全责任制，努力增加绿色优质农产品供给，大力发展绿色种植、绿色养殖等高效生态循环农业，新建省级粮食生产功能区8万亩、省级农业产业集聚区1个、省级特色农业强镇2个、设施农业1万亩、绿色农产品基地1万亩、现代农业科技示范基地30个。创建畜牧业绿色发展示范县1个、省级示范性美丽牧场15家。培育现代农业经营主体，新增示范性家庭农场30家、上市挂牌企业2家。办好义乌森博会、华东农交会、中国苗交会、磐安药博会等农业展会。

促进农村转美。创建美丽乡村风景线9条、精品村45个、秀美村90个、3A级以上景区村20个，新建或改建旅游厕所185个，治乱美化创建达标村95%以上，生活污水治理、生活垃圾分类处理实现行政村全覆盖。

推动农民转行。深化农村土地承包经营权、宅基地用益物权、集体经济收益权“三权到人、权跟人走”改革，完成农村土地确权登记颁证。加强农民职业技能培训和创业扶持，深入实施集体经济薄弱村消除三年行动计划和低收入农户收入倍增计划，培训农村实用人才2万人，建成电商特色镇10个、电商专业村100个，低收入农户人均可支配收入增长10%以上。

　（八）以基本公共服务均等化提升群众获得感

　　鼓励创业、扩大就业、促进社保全覆盖。支持各类人才在金创业、来金创业。做好高校毕业生、就业困难人员、农村转移劳动力等重点群体就业工作，新增城镇就业5.8万人以上，城镇登记失业率控制在3.5%以内。完成市民卡发放100万张。加大社保扩面力度，稳步提高退休人员基本养老金、城乡居民基础养老金和城乡低保标准。加快发展养老服务业，加强医养结合养老服务机构建设，形成城乡居家养老20分钟服务圈。实现大病医疗保险市级统筹，完善社会救助体系，关爱弱势群体，加强城市无障碍设施建设。

加快教育改革发展。制定学前教育第三轮行动计划，实施学前教育、义务教育扩容工程，完成23所薄弱学校提标改造，加强师资队伍和校园文化建设，争创省教育基本现代化县2个。推进中高职融合发展，重视发展特殊教育，积极支持浙师大、金华职业技术学院、上海财大浙江学院、义乌工商学院等在金高校加快发展，做好高水平应用型高校筹建工作。

深化医药卫生体制综合改革。强化“双下沉、两提升”工作，推进分级诊疗，加强社区卫生服务中心、乡镇卫生院和村级卫生服务组织建设，推进县域心电、影像、检验、远程会诊、消毒供应等五大共享中心建设。加快市人民医院、妇幼保健院、中心血站等重点项目建设。加强慢性病防治和传染病防控，大力发展中医药事业。推进医药价格改革。提升计生服务水平。

丰富群众文化生活。实施优秀传统文化传承发展工程，完善基本公共文化服务体系。建成市科技文化广场，推进非遗馆和数字文化馆、图书馆、博物馆建设，发展收藏文化事业，推动农村文化礼堂建管用育一体化。加强对外文化交流，打造婺剧文化金名片。争创全国文明城市提名资格城市。积极发展体育事业和全民健身运动。

深入推进平安金华、法治金华、信义金华建设。健全立体化治安防控体系，防范和打击各类违法犯罪活动，重视网络安全，维护国家安全。落实安全生产“党政同责、一岗双责”，积极创建国家食品安全城市，打造“互联网+菜篮子”全链条可监控可追溯体系。提升防汛防台、防灾减灾、应急管理能力，抓好重要地质灾害隐患点治理、城乡危旧房整治。加强和改进信访、矛盾调处、8890便民服务、12345政务服务等工作，抓好乡镇（街道）“四个平台”建设，完善社会信用体系，推进“法治+德治+自治”综合治理。争创全国双拥模范城“四连冠”，创建东阳、永康省级军民融合创新示范区，支持部队做好全面停止有偿服务工作。

各位代表，今年我们将按照“群众提、代表定、政府办”的要求，切实办好十方面民生实事。同时，支持工会、共青团、妇联、科协等群团组织改革工作，重视做好民族、宗教、对台、民防、气象、统计、档案、地方志、残疾人和关心下一代等工作。

四、切实加强政府自身建设

　　贯彻落实市委关于全力打造“金华铁军”的要求，以更高标准、更严要求、更实作风，加强政府自身建设，全面提升政府系统的执行力、战斗力和公信力。

（一）提能增效，建设服务政府。以“最多跑一次”倒逼“放管服”改革，建设“互联网+政务服务”先行区，走好网上群众路线，及时回应社会关切，当好“店小二”，优化服务供给，着力构建“亲”“清”新型政商关系，大力营造亲商、安商、富商氛围，依法平等保护各类产权。

（二）担当有为，建设责任政府。以时不我待、舍我其谁的担当精神，当工匠、钉钉子，提高执行力、增强公信力。建立重点工作清单制度，强化首问负责制，健全容错纠错机制，严格绩效管理，严肃追责问责，使政府各项工作有清单、有督查、有考核、有问责，营造真抓实干、狠抓落实的浓厚氛围。

（三）依法行政，建设法治政府。切实把各项行政行为纳入法治轨道，做到授予有据、行使有规、施政有信、监督有效。认真执行重大行政决策程序规定，依法民主科学决策。深化行政执法和行政复议体制改革，严格规范公正文明执法。主动接受人大依法监督、政协民主监督以及监察监督、司法监督，加强审计监督，畅通社会和舆论监督渠道。全面推进政务公开工作，让权力公开透明、阳光运行。

（四）严守规矩，建设廉洁政府。牢固树立政治意识、大局意识、核心意识、看齐意识，严守政治纪律和政治规矩，严格落实中央八项规定精神，严防“四风”问题反弹回潮。健全廉政风险防控机制，严控“三公”经费支出，严肃查处各类违纪违法案件。切实治理庸政懒政怠政现象，从群众急切盼望的事情抓起，从社会反映集中的问题改起，用政府“紧日子”换来群众“好日子”。

各位代表，蓝图已绘就，关键在实干。让我们更加紧密地团结在以习近平同志为核心的党中央周围，在省委、省政府和市委的坚强领导下，撸起袖子加油干，扑下身子抓落实，为高水平全面建成小康社会、谱写现代化都市区建设新篇章而努力奋斗！
